# Rethinking the role of sham TMS

**DOI:** 10.3389/fpsyg.2015.00210

**Published:** 2015-02-26

**Authors:** Felix Duecker, Alexander T. Sack

**Affiliations:** ^1^Department of Cognitive Neuroscience, Faculty of Psychology and Neuroscience, Maastricht University, Maastricht, Netherlands; ^2^Maastricht Brain Imaging Center, Maastricht University, Maastricht, Netherlands

**Keywords:** transcranial magnetic stimulation, sham TMS, side effects, placebo, control condition

## Abstract

Sham transcranial magnetic stimulation (TMS) approaches are widely used in basic and clinical research to ensure that observed effects are due to the intended neural manipulation instead of being caused by various possible side effects. We here critically discuss several methodological aspects of sham TMS. Importantly, we propose to carefully distinguish between the placebo versus sensory side effects of TMS. In line with this conceptual distinction, we describe current limitations of sham TMS approaches in the context of placebo effects and blinding success, followed by a short review of our own work demonstrating that the sensory side effects of sham TMS are not unspecific as often falsely assumed. Lastly, we argue that sham TMS approaches are inherently insufficient as full-fledged control conditions as they fail to demonstrate the specificity of TMS effects to a particular brain area or time point of stimulation. Sham TMS should therefore only complement alternative control strategies in TMS research.

## INTRODUCTION

Transcranial magnetic stimulation (TMS) is a non-invasive brain stimulation technique that is widely used as a research tool in the field of neuroscience and has great potential as a treatment option in patients with neurological or psychiatric disorders (Hallett, 2000; Pascual-Leone et al., 2000; Walsh and Cowey, 2000; Sack, 2006). Generally speaking, TMS can influence neuronal activity by exposing the brain to a rapidly changing magnetic field; leading to a variety of immediate and longer-lasting effects that depend on the stimulation parameters. Irrespective of how TMS is applied, however, the intended neural effects of TMS are always accompanied by various psychological as well as sensory side effects. Due to the current flow in the TMS coil, strong vibrations occur that produce a distinct clicking sound with a loudness that is difficult to fully attenuate with passive hearing protection (Counter and Borg, 1992; Rossi et al., 2009; Dhamne et al., 2014). Moreover, the magnetic field induced by the TMS coil also stimulates the skin resulting in somato-sensory effects, and peripheral nerve stimulation often evokes strong twitches of facial muscles. All these effects are obviously unintended in the context of brain stimulation and pose methodological challenges that have to be dealt with.

The problems associated with the clicking sound of the TMS coil and skin stimulation were already recognized in the early days of TMS, leading to a strong awareness of the need for control conditions in TMS research. In general terms, one has to ensure that observed effects of TMS are indeed caused by the direct neural effects instead of being produced by other indirect effects of TMS. Importantly, there are two distinct issues at play that need to be considered separately. On the one hand, the presence of auditory and somato-sensory perception during the application of TMS might be distracting, lead to multi-sensory experiences, trigger shifts of spatial attention, or have an effect on alertness. We will refer to these effects as the sensory side effects of TMS. These effects are problematic as they might interact with relevant aspects of the experimental paradigm. On the other hand, the presence of auditory and somato-sensory perception, but also other TMS-related cues such as positioning a TMS coil on the head, can suggest to participants and patients that TMS pulses are applied to their head. This can result in placebo effects, that is, produce behavioral and physiological changes due to the *belief* that one is undergoing brain stimulation leading to specific expectations of behavioral or cognitive changes that are unrelated to the direct neural effects of TMS. We will refer to these effects as the placebo effects of TMS. Together, these considerations motivated the introduction of sham TMS approaches that, ideally, reproduce the sensory effects of active TMS but lack its direct neural effects and can therefore serve as control conditions for both placebo and sensory side effects of TMS.

In the following, we first describe common sham TMS approaches and address several issues revolving around the use of sham TMS in basic and clinical research. To begin with, we discuss whether sham TMS is sufficiently similar to active TMS so that both result in the same beliefs regarding the stimulation effects, that is, whether sham TMS is a good control for the placebo effects of TMS. We then focus on the sensory side effects of TMS on behavior and review our own empirical work that revealed surprisingly specific effects of sham TMS. Based on these studies, we provide empirical evidence that allows evaluating the quality of sham TMS as a control approach in TMS research. Lastly, we argue that the concept of control conditions in TMS research is not restricted to the placebo and sensory side effects of TMS. In the light of this view, sham TMS is seriously limited and its use has to be carefully balanced with other methodological considerations.

## SHAM TMS APPROACHES

The term “sham TMS” is not without ambiguity as it may refer to any approach that aims at mimicking the auditory and/or somato-sensory effects of active TMS without actual stimulation of the brain. There is a surprising variety of sham TMS approaches but, generally speaking, they can be grouped in two distinct categories.

On the one hand, there are approaches that make use of a regular TMS coil that is tilted so that an edge remains in contact with the head. As a consequence, a sham TMS pulse produces a clicking sound that is very similar to an active TMS pulse and, depending on the geometry and orientation of the TMS coil, the magnetic field can still be sufficiently strong to result in somato-sensory effects and even peripheral nerve stimulation (Loo et al., 2000; Lisanby et al., 2001). Obviously, it is rather difficult to determine whether or not there is residual brain stimulation in such cases, essentially requiring a trade-off between matching the sensory side effects of active TMS and ensuring the absence of neural effects.

On the other hand, there are purpose-built sham TMS coils that resemble regular TMS coils but are equipped with a magnetic shield that attenuates the magnetic field. As a consequence, these sham TMS coils can be positioned exactly like an active TMS coil resulting in a very good approximation of the auditory effects. In contrast to the sham TMS approach outlined above, the strong attenuation of the magnetic field ensures that no brain stimulation occurs. However, this also abolishes the somato-sensory effects and peripheral nerve stimulation that occurs during the application of active TMS. In order to counteract this limitation, surface electrodes can be used for electrical stimulation of the skin time-locked to a TMS pulse, with the idea to render the somato-sensory effects of sham and active TMS indistinguishable.

From this description alone one might argue that a sham TMS coil in combination with electrical stimulation is the best sham TMS approach. It is by far the most sophisticated setup and promises to achieve the best approximation of the auditory and somato-sensory effects of active TMS while ensuring that no brain stimulation occurs. Yet, no sham TMS approach should either be discarded or embraced based on theoretical grounds as the effectiveness of different sham TMS approaches essentially is an empirical question.

## THE PLACEBO EFFECTS OF TMS

It is rather obvious that the application of TMS is the critical manipulation in any TMS study, both in basic and clinical research. Participants and patients typically receive considerable information about TMS in advance and they inevitably speculate about its effects. The occurrence of placebo effects therefore is at least plausible and poses a threat to TMS studies. To be on the safe side, a general requirement for sham TMS approaches should therefore be that they are sufficiently similar to active TMS so that they result in the same beliefs regarding the stimulation effects. Specifically, participant and patients should not be able to infer that sham TMS is applied, that is, they should be blind to the stimulation condition. Moreover, even if blinding is successful, the overall experience (somato-sensory effects, muscle twitches, discomfort, etc…) during sham TMS should be as similar as possible to active TMS because any mismatch between them could change the beliefs about the effectiveness of stimulation, potentially affecting the magnitude of the placebo effect.

In basic TMS research, there is a surprising lack of studies investigating placebo effects. In fact, a PubMed search (keywords: transcranial magnetic stimulation AND placebo) identified only one original research article in a non-clinical context. This study assessed the performance on a motor learning task with or without sham stimulation and found no indication for a placebo effect (Jelić et al., 2013). The issue has thus been largely ignored but given that TMS is widely used to investigate all kinds of mental phenomena, this null result should not be taken as an indication that placebo effects are not a concern in basic research. Similarly, the success of blinding participants and experimenters with respect to the stimulation condition (active vs. sham) has hardly received any attention. Although there is an abundance of sham-controlled TMS studies published over the years, it is not common practice to report information on blinding success. There is only anecdotal evidence with a somewhat amusing case where experienced researchers failed to notice that they were trying to determine the motor threshold for more than an hour with a sham TMS coil (Segev and Bloch, 2013). From our own experience, most researchers seem to be very aware of the limitations of most sham TMS approaches and rather not expose a possible confound of their study by not collecting any data on blinding success and how the stimulation was experienced. We would like to emphasize the importance of obtaining such empirical data because explicitly facing this issue will eventually help the entire field to progress to higher-quality sham TMS approaches.

The considerations above are in contrast to clinical TMS research where the issue of placebo effects and the importance of blinding success have received more attention (Davis et al., 2013). To begin with, placebo effects have indeed been observed in many TMS treatment studies for various brain-related disorders including major depression (Brunoni et al., 2009), epilepsy (Bae et al., 2011), obsessive-compulsive disorder (Mansur et al., 2011), and Parkinson’s disease (Okabe et al., 2003). Similar to basic research, data on blinding success is rarely reported but there are two recent reviews that addressed this issue albeit based on a rather small number of studies (Broadbent et al., 2011; Berlim et al., 2013). In general, both reviews did not report significant differences between sham and active TMS groups in correctly guessing their treatment allocation but studies sometimes failed to maintain blinding integrity. Moreover, given that less than 15% of all randomized sham-controlled clinical trials reported data on blinding success, it remains unclear whether researchers often withhold this information in case of blinding failure (Broadbent et al., 2011). Yet, these data suggest that blinding of patients works relatively well in clinical settings where between-subject designs are commonly employed. In basic research, however, within-subject designs are much more common and, in our view, it is therefore questionable how well these results generalize to other research settings. Participants that undergo both active and sham TMS throughout an experiment are in a much better position to tell stimulation conditions apart compared to patients that are assigned to one particular treatment group.

The blinding success of sham TMS approaches first and foremost is a technological challenge as it depends on mimicking the acoustic and somato-sensory effects of active TMS while ensuring that no brain stimulation occurs. From this perspective, the use of surface electrodes for skin stimulation in combination with a shielded TMS coil seems the current gold standard. The critical question thus is whether blinding success can be achieved under these optimal conditions. Several very similar sham TMS setups have been developed over the years and their perceptual effects and blinding success have been evaluated (Rossi et al., 2007; Arana et al., 2008; Borckardt et al., 2008; Mennemeier et al., 2009; Sheffer et al., 2013). The general finding of these studies is that electrical stimulation of the skin results in somato-sensory effects that are very similar to active TMS if the stimulation intensity is individually calibrated (Arana et al., 2008; Borckardt et al., 2008; Mennemeier et al., 2009). However, the skin sensation is more electric so that experienced participants might be able to discriminate between active and sham TMS. And indeed, naïve participants have been found to mistake sham TMS for active TMS under such conditions (Rossi et al., 2007; Mennemeier et al., 2009; Sheffer et al., 2013) whereas experienced participants can tell them apart (Rossi et al., 2007; Mennemeier et al., 2009). Taken together, these results indicate that sham TMS approaches require further developments but might suffice for clinical applications where patients are generally naïve to differences between active and sham TMS. Yet, as noted before, successful blinding does not necessarily imply that placebo effects are fully controlled for because patients might still have different beliefs about the effectiveness of stimulation based on the intensity of the experience which can be difficult to match across conditions.

Finally, we would like to point out that sham TMS approaches using electrical stimulation thus far aimed at *mimicking* the somato-sensory effects of active TMS. The results described above show that this is difficult to achieve. In our view, this might actually not be necessary because control for placebo effects only requires active and sham TMS to be *indistinguishable*. Therefore, an interesting alternative to current approaches could be to apply electrical stimulation also during active TMS, potentially *masking* the differences between them.

## THE SENSORY SIDE EFFECTS OF TMS

Whenever TMS is applied during the execution of a task, it is indispensable to ensure that the observed behavioral effects of TMS are caused by the intended neural manipulation instead of being produced by the clicking sound of the TMS coil or sensations on the head. On a conceptual level, this has long been recognized but there is a surprising lack of empirical knowledge regarding how these sensory side effects of TMS influence task performance. Even though there is an abundance of studies on multisensory integration and its role in attention and performance (e.g., Niemi and Näätänen, 1981; Stein et al., 1989; Spence and Driver, 1997), the significance of such effects in the context of TMS experiments is largely unknown. It therefore remains unclear what TMS control strategies actually have to control for, making it difficult to evaluate their adequacy. Recently, we have started to build an empirical foundation of control strategies in TMS research (Duecker et al., 2013; Duecker and Sack, 2013). Reversing the logic of usual TMS experiments, we used a sham TMS coil to investigate the sensory side effects of TMS on behavior without potential confounding due to neural effects of TMS.

In our first experiment (Duecker and Sack, 2013), we applied single-pulse sham TMS either over the left or right hemisphere prior to presentation of a visual target in the left or right hemifield. The critical finding was that these lateralized sham TMS pulses caused automatic shifts of spatial attention toward the position of the TMS coil thereby facilitating target detection in the corresponding hemifield. This shows that the sensory side effects of TMS are highly specific and can influence task performance dependent on the exact location from which the sound of the TMS pulse originates. In our second experiment (Duecker et al., 2013), we applied either single-pulse sham TMS or active TMS over the vertex, a stimulation site where neural effects are typically not expected, and explored potential changes in task performance across a broad range of pre- and post-stimulus time windows in the context of a detection task and angle judgment task. Again, we found highly specific sensory side effects that were dependent on a complex interplay of the task, type of stimulation, and time point of stimulation. Importantly, the effects of sham and active TMS were almost identical in the context of this particular experiment showing that sham TMS is capable of approximating the sensory side effects of active TMS. However, small differences between sham and active TMS were still present, most likely due to the lack of somato-sensory effects during sham TMS.

The experiments outline above revealed surprisingly specific sensory side effects of TMS but are obviously only a first step as these effects most likely vary considerably across tasks and may depend on parameters not yet considered. It therefore seems highly desirable to take advantage of existing and future sham TMS data to extend the empirical knowledge regarding the sensory side effects of TMS. In this sense, sham TMS is not simply one of many control strategies but informative in itself with its unique contribution to the development of TMS methodology.

## SHAM TMS AS CONTROL CONDITION

Sham TMS approaches are widely used as control conditions in TMS research. Keeping their current limitations in mind, they still seem particularly well-suited to control for the sensory side effects of TMS because they allow using identical stimulation parameters as during active TMS. That is, a sham TMS coil can be positioned over the same brain area and the sham TMS pulses can be applied at the same time points during task execution. Importantly, we have shown above that these stimulation parameters influence task performance. It follows that observed differences between TMS target sites or time points do not necessarily arise from the neural effects of TMS. Alternative control approaches based on the application of active TMS therefore generally fail in case of highly specific sensory side effects of TMS simply because either the stimulation site or stimulation time point is not kept constant. However, a noteworthy exception is the manipulation of TMS coil orientation which results in changes of the induced electric field without acoustic or somato-sensory differences between TMS conditions (Thielscher et al., 2011). However, it is by no means guaranteed that this approach always results in differential effects and it requires detailed knowledge regarding optimal TMS coil positioning. We therefore argue that sham TMS approaches have distinct advantages in controlling for the sensory side effects of TMS and they will further improve as advanced methods for mimicking/masking the somato-sensory aspects of active TMS are developed.

Considerations regarding control conditions in TMS research are not restricted to the side effects of TMS. Most TMS experiments aim at revealing that stimulation of a *particular* brain area has specific behavioral and/or physiological consequences. Similarly, TMS can be used to show that a brain is functionally relevant at a *particular* point in time during task execution. In order to make such claims, it is necessary to show that the same effects do not occur when stimulating *another* brain area or time point. In this sense, TMS experiments always require an active TMS control condition, and sham TMS approaches can never be sufficient as they fail to demonstrate such specificity. We therefore argue for a complementary use of sham TMS approaches, limited to controlling for the sensory side effects of TMS, alongside with active TMS control strategies. A typical TMS experiment should therefore consist of multiple stimulation sites or stimulation time points using active TMS. Sham TMS can then be used as an orthogonal control condition, essentially copying all stimulation parameters. Importantly, this also implies using sham TMS over each brain area where active TMS is applied in order to make sure that all stimulation sites have a proper control condition for the sensory side effects of TMS. As noted before, whether or not such tight control is necessary depends on the specificity of these effects, and eventually practical considerations might demand compromises in the design of an experiment.

## CONCLUSION

Sham TMS approaches serve multiple purposes in TMS research as they are used to control for the placebo and sensory side effects of TMS. Issues regarding placebo effects and the blinding success of sham TMS are mostly technological challenges and we have outlined current problems and a possible solution. In the broader context of control strategies, sham TMS can be used to identify and control for the sensory side effects of TMS on behavior. Moreover, we have argued for a complementary role of sham TMS as it has its particular strengths but is ultimately insufficient as a full-fledged control paradigm.

### Conflict of Interest Statement

The authors declare that the research was conducted in the absence of any commercial or financial relationships that could be construed as a potential conflict of interest.
